# LINC00467 Is Upregulated by DNA Copy Number Amplification and Hypomethylation and Shows ceRNA Potential in Lung Adenocarcinoma

**DOI:** 10.3389/fendo.2021.802463

**Published:** 2022-01-13

**Authors:** Wen Wang, Hao Bo, Yumei Liang, Guoli Li

**Affiliations:** ^1^ Department of Cardio-Thoracic Surgery, Hunan Provincial People’s Hospital (The First-Affiliated Hospital of Hunan Normal University), Changsha, China; ^2^ Clinical Research Center for Reproduction and Genetics in Hunan Province, Reproductive and Genetic Hospital of CITIC-Xiangya, Changsha, China; ^3^ Department of Nephrology and Laboratory of Kidney Disease, Hunan Provincial People’s Hospital (The First-Affiliated Hospital of Hunan Normal University), Changsha, China; ^4^ Changsha Clinical Research Center for Kidney Disease, Changsha, China; ^5^ Hunan Clinical Research Center for Chronic Kidney Disease, Changsha, China

**Keywords:** LINC00467, lung adenocarcinoma, DNA copy number, DNA methylation, CeRNA

## Abstract

Lung adenocarcinoma (LUAD) is the most common histological lung cancer, and it is the leading cause of cancer-related deaths worldwide. Long noncoding RNAs (lncRNAs) have been implicated in tumorigenesis. LINC00467 is a novel lncRNA that is abnormally expressed in several cancer types including LUAD. However, its function and regulatory mechanism in LUAD progression remain unclear. In this study, based on The Cancer Genome Atlas data mining, we demonstrated that DNA copy number amplification and hypomethylation was positively correlated with LINC00467 expression in LUAD. In addition, DNA copy number amplification was significantly associated with distant metastasis, immune infiltration and poor survival. Microarray analysis demonstrated that LINC00467 knockdown in the LUAD A549 cell line led to a distinct microRNA expression profile that impacted various target genes involved in multiple biological processes. This finding suggests that LINC00467 may regulate LUAD progression by functioning as a competing endogenous RNA (ceRNA). Finally, we constructed a ceRNA network that included two microRNAs (hsa-miR-1225-5p, hsa-miR-575) and five mRNAs (BARX2, BCL9, KCNK1, KIAA1324, TMEM182) specific to LINC00467 in LUAD. Subsequent Kaplan-Meier survival analysis in both The Cancer Genome Atlas and Gene Expression Omnibus databases revealed that two genes, *BARX2* and *BCL9*, were potential prognostic biomarkers for LUAD patients. In conclusion, our data provide possible mechanisms underlying the abnormal upregulation of LINC00467 as well as a comprehensive view of the LINC00467-mediated ceRNA network in LUAD, thereby highlighting its potential role in diagnosis and therapy.

## Introduction

Lung adenocarcinoma (LUAD) is the most common histological lung cancer; it is the leading cause of cancer-related deaths and the second most commonly diagnosed cancer worldwide (1). Despite advancements made in targeted therapies and immunotherapies, the overall survival rate of LUAD patients remains low (2). A major challenge in furthering LUAD treatment is the lack of effective targets and biomarkers. Accordingly, novel molecular targets and diagnostic or therapeutic biomarkers are urgently needed.

Long non-coding RNAs (lncRNAs) are transcripts that are longer than 200 nucleotides. As they lack or have limited protein-coding abilities, lncRNAs were traditionally regarded as junk transcripts with no function (3). However, recent studies have found that they play key roles in multiple biological processes and pathological conditions by regulating gene expression at different levels (4). Importantly, lncRNAs can facilitate tumor progression either by promoting oncogene function or by inhibiting tumor suppressor genes (5). A common molecular regulatory mechanism of lncRNAs is competitive endogenous RNA (ceRNA); it has been observed in several cancer types including LUAD (6). LncRNAs have been suggested to compete with microRNAs (miRNAs) as a molecular sponge leading to the degradation of target mRNAs (6). To date, over 10,000 lncRNAs have been identified in the human genome (7). Nevertheless, only a small proportion of them have been analyzed for biological function (3). Consequently, additional research is required to advance our understanding of their function.

Long intergenic non-protein coding RNA 467 (LINC00467) is a recently identified lncRNA (8). Its expression is altered in several cancer types, including both solid tumors (9–15) and hematologic malignancies (16). In LUAD, LINC00467 expression has been reported to be upregulated, and its high expression contributes to tumorigenesis by promoting cell proliferation or stemness (17–21). However, the mechanism underlying its abnormal upregulation and its prognostic significance in LUAD remains elusive.

To deepen our understanding of LINC00467 function in LUAD, in this study, we performed data mining on The Cancer Genome Atlas (TCGA) as well as microarray analysis to identify upstream regulation mechanisms and downstream network of LINC00467. We hypothesized that DNA copy number alterations and promoter methylation may be two important mechanisms contribute to LINC00467 expression, and LINC00467 could widely impact on biological functions as a ceRNA. Our findings indicate that LINC00467 may be a potential target and diagnosis or prognosis biomarker for LUAD.

## Materials and Methods

### Cell Culture and Reagents

The LUAD cell line A549 was obtained from American Type Culture Collection and confirmed to be free of *Mycoplasma* using the LookOut Mycoplasma PCR Detection Kit (Sigma). Cells were grown in RPMI1640 medium with 5% fetal bovine serum (Hyclone/Gemini) and 1% penicillin/streptomycin (Gibco); they were maintained at 37°C in a humidified incubator with 5% CO_2_. 5-Azacytidine (5-AZA) was purchased from Selleck.

### Cell Transfection

A549 cells were passaged onto culture plates until 70–80% confluence was reached. The cells were transfected with small interfering RNA (siRNA) against LINC00467 (si-LINC00467) using Lipofectamine 2000 (Invitrogen) in accordance with the manufacturer’s instructions. Empty vectors were used as the negative control (NC). SiRNAs were purchased from Ribobio (Guangzhou, China). The silencing effect of si-LINC00467 was confirmed in our previous study (21). The cells were tested after 48h of transfection.

### Analysis of miRNA Expression Profiles

MiRNA profiling was performed on six A549 cell samples. Of these, three samples were LINC00467 knockdown, and three were not. The experiments were conducted by Shanghai OE Biotech Co. Ltd. using the Affymetrix miRNA 3.0 Array system (Affymetrix). Briefly, RNA was labeled with the FlashTag™ Biotin HSR RNA Labeling Kit for poly(A)-tailing according to the manufacturer’s protocol. Afterwards, the labeled samples were hybridized with the Affymetrix GeneChip miRNA 3.0 Array following the manufacturer’s instructions. This microarray contains 19,724 total mature miRNA probe sets, including 1,733 mature human miRNAs and 153 miRNAs from other organisms.

### Reverse Transcription Quantitative Real-Time Polymerase Chain Reaction Assays

After 48 h of 5-azacytidine treatment, the total RNA in the A549 cells was isolated using Trizol reagent (Thermo Fisher Scientific). cDNA synthesis was conducted using a SuperScript II RT-PCR kit (Life Technologies), while quantitative PCR was performed using the SYBR Green Master Mix (Life Technologies) with the QuantStudio 3 Real-Time PCR System (Life Technologies) according to the manufacturer’s instructions. The primer sequences are as follows: LINC00467 F: 5’-TCGTCTTCAGGAAGCCAGAC-3’ and R 5’-TGGAAATCAAAAGGGTCAGC-3’. GAPDH F: 5’-GGAGCGAGATCCCTCCAAAAT-3′ and R 5′-GGCTGTTGTCATACTTCTCATGG-3′. LINC00467 expression was normalized using glyceraldehyde 3-phosphate dehydrogenase (GAPDH) as a reference. The relative expression level of LINC00467 was calculated using the 2^−ΔΔCt^ method.

### Bioinformatics Analysis

LINC00467 DNA copy number alteration data and patient clinical data were downloaded from the TCGA data portal (22) (website: https://www.cbioportal.org/; dataset: TCGA, PanCancer Atlas). The correlation between LINC00467 DNA copy number alterations and immune cell infiltration was evaluated in TIMER2.0 (23) (http://timer.cistrome.org/). The correlations between molecular marker expression in immune cells and patient prognosis were evaluated using GEPIA2 (24) (http://gepia2.cancer-pku.cn/#index). GEPIA2 was also used to compare multiple genes between LUAD tumor tissues and normal tissues as well as the correlations between their expression levels. Heat maps of LINC00467 expression and promotor DNA methylation in the TCGA database were obtained from the UCSC Xena web-based tool (25). PITA (26), MicroRNA.org (27), and TargetScan (28) (https://xena.ucsc.edu/) databases were used to identify miRNA-mRNA interaction pairs. Survival analyses were performed using the Kaplan-Meier plotter (29) (http://kmplot.com/analysis/). The binding sites between LINC00467 and miRNAs was predicted by DIANA-LncBase v2 (30) (https://carolina.imis.athena-innovation.gr/diana_tools/web/index.php?r=lncbasev2%2Findex-predicted).

### Statistical Analysis

The experimental data were analyzed using GraphPad Prism (GraphPad). Student’s *t*-test was used to compare between two groups, while comparisons among multiple groups were performed using one-way analysis of variance. Survival curves were compared using the log-rank test. Percentage data was compared *via* the chi-square test. Finally, correlations were performed using Pearson’s correlation coefficient. *P* < 0.05 was considered statistically significant.

## Results

### LINC00467 DNA Copy Number Alterations Were Significantly Associated With LINC00467 Expression and Immune Infiltration and Predicted Poor Prognosis in LUAD

**Figure 1:**
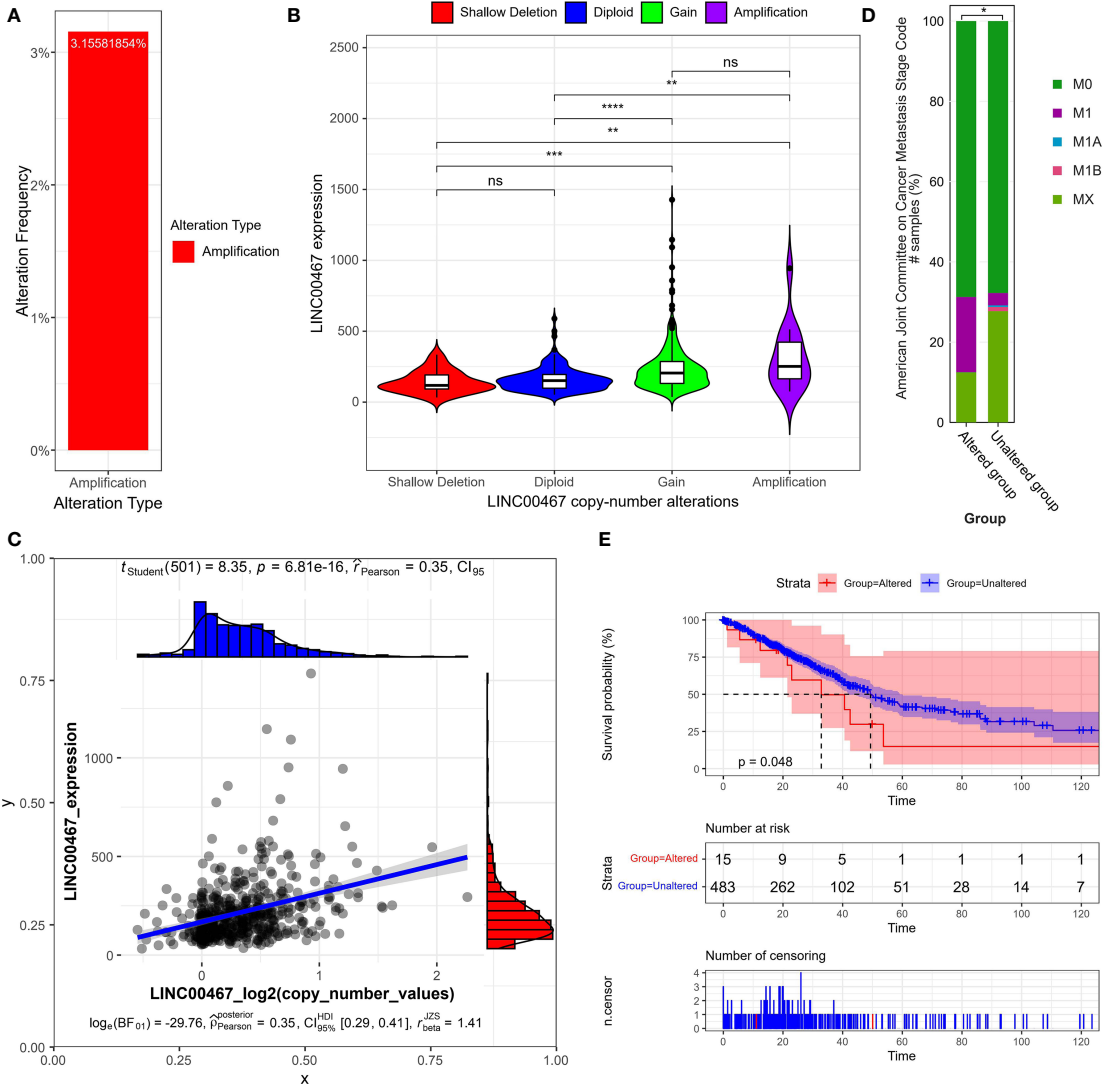
LINC00467 expression correlates with DNA copy number alterations in LUAD. **(A)** The percentage of patients with DNA copy number amplification of LINC00467 in the LUAD cohort (The Cancer Genome Atlas [TCGA], PanCancer Atlas). **(B)** LINC00467 expression in LUAD patients across different types of DNA copy number alterations. As defined by GISTIC2.0, Deep Deletion indicates a deep loss, possibly a homozygous deletion; Shallow Deletion indicates a shallow loss, possibly a heterozygous deletion; Diploid indicates normal; Gain indicates a low-level gain (a few additional copies, often broad); Amplification indicates a high-level amplification (more copies, often focal. Please see: https://docs.cbioportal.org/1.-general/faq#dna-mutations-copy-number-and-fusions. **P < 0.01; ***P < 0.001; ****P < 0.0001; ns, no significance. **(C)** Correlation between LINC00467 expression and DNA copy number. **(D)** The percentage of patients in different metastasis stages with altered versus unaltered LINC00467 DNA copy numbers. According to “The Eighth Edition Lung Cancer Stage Classification” (31): M0 indicates no distal metastasis; M1A indicates intrathoracic metastasis or pleural effusion or pericardial effusion or contralateral lung nodules/pleural nodules; M1B indicates single extrathoracic metastasis in a single organ; MX indicates metastasis cannot be measured. *P < 0.05. **(E)** Overall survival of patients with altered versus unaltered LINC00467 DNA copy numbers. The horizontal axis indicates the overall survival time in months, and the vertical axis indicates the survival rate.

To explore the mechanisms underlying LINC00467 upregulation in LUAD, we retrieved online sequencing data from the TCGA LUAD cohort (Lung Adenocarcinoma TCGA PanCancer), which comprised of 566 patients. Altogether, 16 patients had DNA copy number amplification, which accounted for 3.16% of patients with copy number alterations data (N=507) in the cohort (**Figure 1A**). We first analyzed LINC00467 expression across four different types of putative copy number alterations from Genomic Identification of Significant Targets in Cancer (GISTIC). The results indicated that type amplification and gain were associated with higher LINC00467 expression relative to type shallow deletion or diploid (**Figure 1B**). We also observed a positive correlation between LINC00467 DNA copy number values and LINC00467 mRNA expression (Pearson’s r = 0.35, *p* = 6.81e-16) (**Figure 1C**).

**Figure 2:**
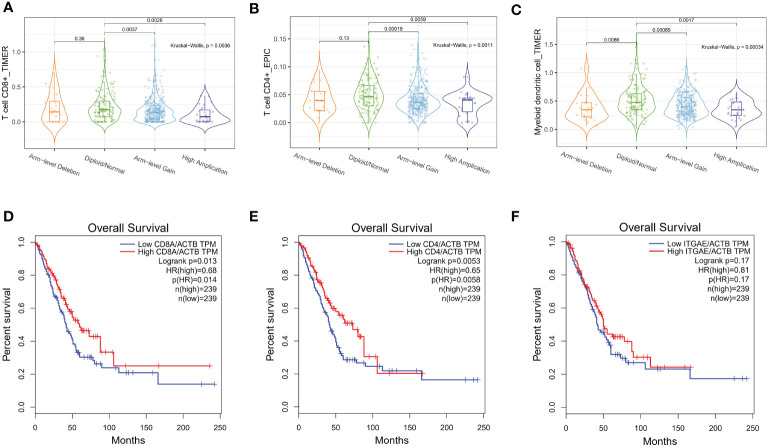
LINC00467 DNA copy number alterations correlate with immune cell infiltration in LUAD. The correlation between LINC00467 DNA copy number alteration and the infiltration levels of CD8^+^ T cells **(A)**, CD4^+^ T cells **(B)**, and dendritic cells (DC) **(C)** in LUAD. The overall survival of patients with low versus high infiltration levels of CD8^+^ T cells **(D)**, CD4^+^ T cells **(E)**, and DC cells **(F)**. ITGAE is a marker of DC cells.

Next, we assessed the independent prognostic value of LINC00467 copy number alterations in terms of tumor metastasis (**Figure 1D**) and overall survival (**Figure 1E**). We found that the LINC00467 copy-number-altered group was associated with more distant metastasis (p <0.05) and poorer overall survival compared to the unaltered group (*p* =0.048). To further investigate the role of LINC00467 in tumor immune response, we analyzed the correlation between LINC00467 DNA copy alterations and the infiltration of three immune cell types using the TIMER algorithm. As shown by the violin plot, we found that LINC00467 DNA copy alterations were correlated with immune infiltration levels of CD8^+^ T cells (*p=*0.0036), CD4^+^ T cells (*p=*0.0011), and dendritic cells (DC) (*p=*0.00034) in LUAD (**Figures 2A–C**). Tumors with alteration status of arm-level gain” and “high amplification had significantly more immune infiltration levels of CD8^+^ T cells (*p=*0.0037 and *p=*0.0026, respectively), CD4^+^ T cells (*p=*0.00019 and *p=*0.0059, respectively), and dendritic cells (DC) (*p=*0.00085 and *p=*0.0017, respectively) when compared to status of “diploid/normal”. Accordingly, the high expression of CD8^+^ T cell (HR=0.68, Logrank *p=*0.013), CD4^+^ T cell (HR=0.65, Logrank *p=*0.0053) and DC molecular markers ITGAE (HR=0.81, Logrank *p=*0.17) was associated with better overall survival in LUAD (**Figures 2D–F**).

### DNA Demethylation Upregulated LINC00467 Expression in LUAD

**Figure 3:**
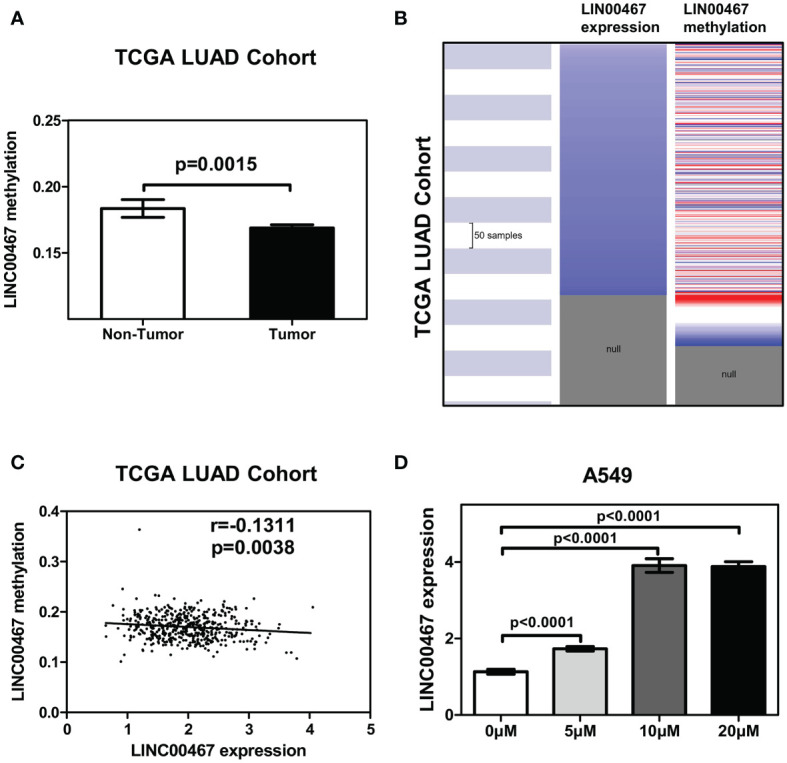
LINC00467 upregulation is modulated by promoter methylation in LUAD. **(A)** Mean methylation levels of the LINC00467 promoter in tumor versus normal tissues in the TCGA LUAD cohort. **(B)** Heatmap of LINC00467 expression and DNA methylation in TCGA LUAD cohort. **(C)** LINC00467 expression negatively correlates with mean LINC00467 promoter methylation levels in TCGA LUAD cohort. **(D)** LINC00467 expression in A549 cells was significantly down-regulated in a dose-dependent manner after 5-azacytidine treatment.

As DNA promoter methylation is another important mechanism modulating gene transcription, we examined the methylation status of the LINC00467 promotor in LUAD using the sequencing data of the TCGA LUAD cohort. We found that the mean level of DNA methylation was significantly lower in tumor tissues than in normal tissues (*p* = 0.0015) (**Figure 3A**). The heatmap indicated that the mean level of LINC00467 methylation was associated with LINC00467 expression in the TCGA LUAD cohort (**Figure 3B**). Additionally, the regression analysis revealed a weak negative correlation between LINC00467 expression and its DNA methylation (Pearson’s *r* = -0.1311, *p* = 0.0038; **Figure 3C**). To further evaluate whether hypomethylation can enhance LINC00467 expression, we performed *in vitro* experiments by adding 5-azacytidine to A549 cells. We found that 5-azacytidine significantly upregulated LINC00467 expression in a dose-dependent manner (**Figure 3D**). Taken together, these results suggest that DNA methylation is an important mechanism modulating LINC00467 expression in LUAD.

### LINC00467 Silencing Revealed a Distinctive miRNA Expression Profile and a Comprehensive miRNAs-mRNAs Network in LUAD

**Figure 4:**
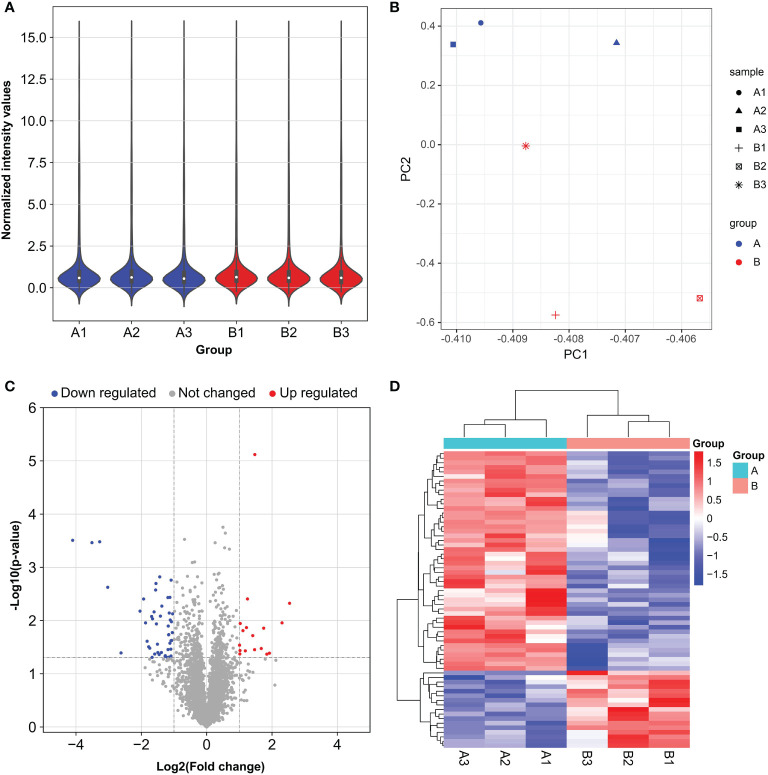
Distinct microRNA (miRNA) expression profiles in A549 cells after LINC00467 knockdown. Group A (A1, A2, A3): control cells; Group B (B1, B2, B3): LINC00467 knockdown cells. **(A)** Violin plots showing the normalized intensity values for miRNA microarray datasets. **(B)** Principal component analysis of the miRNA microarray datasets. **(C)** Volcano plot of miRNA expression. MiRNAs with p-value < 0.05 and |fold change| ≤ 2 were determined as differentially expressed. Up regulated (red) and down regulated (blue) miRNA with twofold change are shown. **(D)** Heat map and hierarchical clustering of the differentially expressed miRNAs. The left vertical axis presents the clusters. The horizontal axis represents the sample groups (control cells in aqua: A1–A3 and LINC00467 knockdown cells in red: B1–B3). Red indicates high relative expression and blue indicates low relative expression.

**Figure 5:**
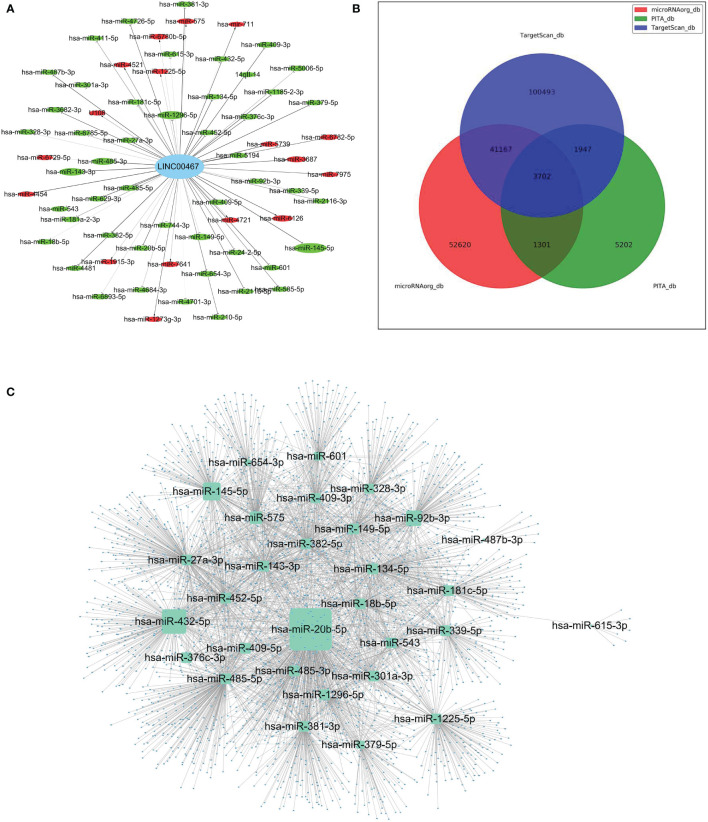
The construction of a LINC00467-mediated competing endogenous RNA (ceRNA) network. **(A)** Global view of LINC00467-mediated network in differentially expressed miRNAs. Down-regulated miRNAs are marked green, while up-regulated miRNAs are marked red. The size of the node represents the degree of differential expression: the higher the degree, the larger the node. **(B)** Potential target genes of the differentially expressed miRNAs as predicted by the three software (PITA, MicroRNA.org, and TargetScan) as represented by a Venn diagram. **(C)** The interaction network of target genes as predicted by all three software and its corresponding miRNAs.

To further study the LINC00467−mediated downstream regulatory mechanism involved in LUAD progression, we analyzed miRNA expression profiles of A549 cells with or without LINC00467 knockdown. After normalization of raw data generated from microarray analysis, the normalized intensity values were on the same level (**Figure 4A**). Principal component analysis shows significant segregation of different group samples (**Figure 4B**). Altogether, 65 known miRNAs were differentially expressed in the LINC00467-knockdown group (Group B) compared to the control group (Group A) (**Figures 4C, D**). Among these differentially expressed miRNAs, 17 were up-regulated, and 48 were down-regulated (**Figure 5A**).

To further investigate the potential role of these differentially expressed miRNAs in LUAD progression, target genes were predicted using three different software (PITA, MicroRNA.org, and TargetScan) (**Figure 5B**). As represented by the Venn diagram, total 3,702 overlapping target genes were predicted by the three software. The relationship of these 3702 mRNAs and its targeted miRNAs were listed in **Supplementary Table 1**. The interaction network of these overlapping target genes and the differentially expressed miRNAs are depicted in **Figure 5C**.

**Figure 6:**
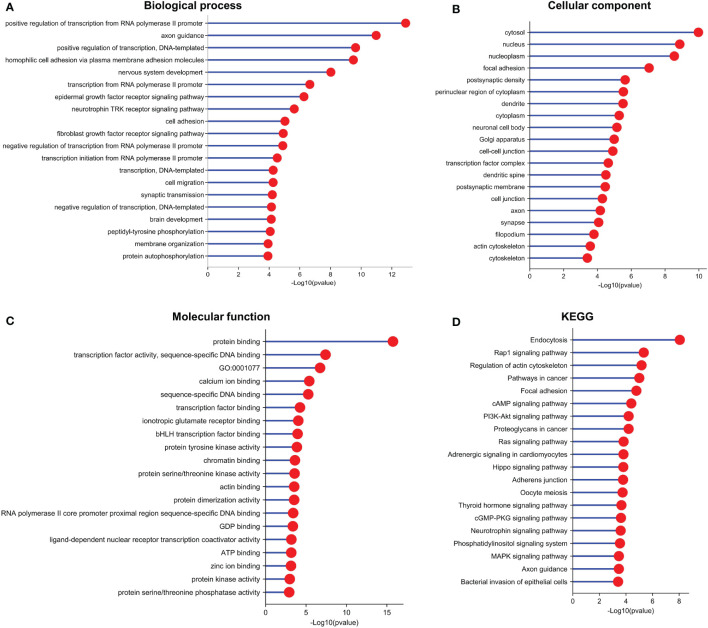
Functional enrichment analysis indicated that the target genes of the differentially expressed miRNAs are involved in multiple pathways and biological processes. **(A)** GO (Gene Ontology)-BP (Biological Process), **(B)** GO-CC (Cell Component), **(C)** GO-MF (Molecular Function), and **(D)** Kyoto Encyclopedia of Genes and Genomes (KEGG) enrichment analysis of target genes of the differentially expressed miRNAs.

To identify the potential biological roles of these target genes in LUAD, Gene Ontology (GO) enrichment and Kyoto Encyclopedia of Genes and Genomes (KEGG) pathway analyses were performed (**Figure 6**). The GO analysis revealed the top 20 GO-enriched Biological Processes (BP) (**Figure 6A**), Cellular Components (CC) (**Figure 6B**), and specific Molecular Functions (MF) (**Figure 6C**) of these target genes. Notably, many of these terms were associated with cell proliferation. The KEGG pathway analysis identified the top 20 enriched pathways of these target genes (**Figure 6D**). The target genes were mainly enriched in “endocytosis pathways”, “Rap1 signaling pathway”, “regulation of actin cytoskeleton”, and “pathways in cancer”. Taken together, these results suggest that LINC00467 regulates LUAD progression by perturbating multiple biological processes.

### Construction of LINC00467-Mediated ceRNA Network Revealing Potential Prognostic Biomarkers for LUAD

**Figure 7:**
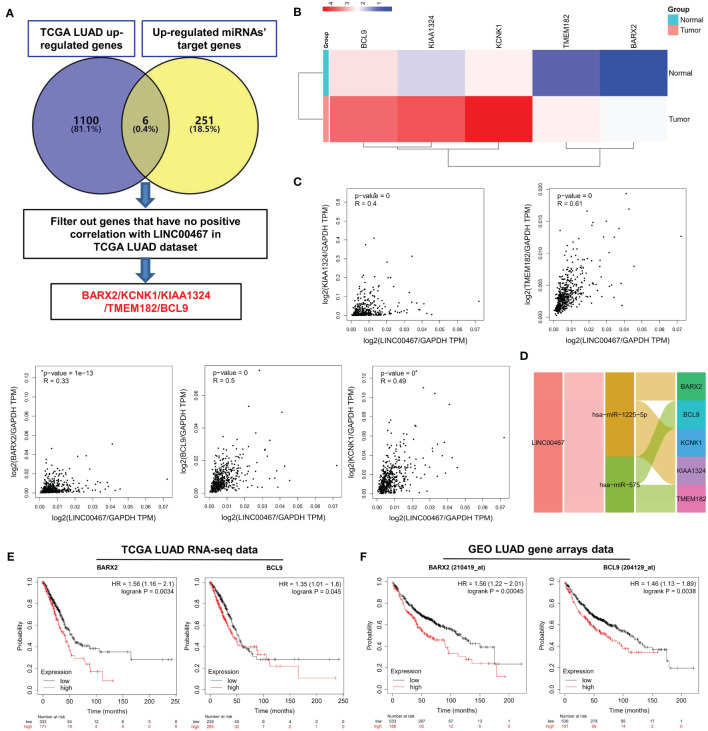
Screening of LUAD prognostic biomarkers in the LINC00467-mediated ceRNA network. **(A)** Flowchart for the identification and analysis of candidate genes. **(B)** Multiple gene comparison analysis was performed using GEPIA2, which revealed the expression of the candidate genes in LUAD tumor versus normal tissues. The density of color in each block represents the median expression value. T indicates tumour and N indicates normal. Use log2(TPM+1) transformed expression data for plotting; **(C)** Correlation analysis was performed *via* GEPIA2, which indicated the relationships between the expression levels of the candidate genes and LINC00467 in LUAD. **(D)** Sankey diagram for the LINC00467 ceRNA network. Each rectangle represents a gene, and the connection degree of each gene is visualized based on the size of the rectangle. Kaplan-Meier curve of the TCGA RNA-seq **(E)** and Gene Expression Omnibus LUAD gene arrays **(F)** data, revealing that *BARX2* and *BCL9* expression correlate with overall survival. The horizontal axis indicates the overall survival time in months, and the vertical axis indicates the survival rate.

To screen for LUAD prognostic biomarkers, we further analyzed the LINC00467 related miRNAs-mRNAs network. As shown in **Figure 7A**, 1,106 up-regulated genes were retrieved from the TCGA LUAD database. Compared to the 257 up-regulated miRNAs’ target genes, six genes were overlapping. After filtering out one gene that was not positively expressed with LINC00467 in the TCGA LUAD dataset, five candidate genes remained. These genes were *BARX2*, *KCNK1*, *KIAA1324*, *TMEM182*, and *BCL9*. Heat map shows that the mRNA expression of these candidate genes in LUAD tumor tissues were all higher than in normal tissues (**Figure 7B**). The positive corrections between the expression of the candidate genes (Pearson’s r = 0.4, *p* = 0, for *KIAA1324*; Pearson’s r = 0.61, *p* = 0, for *TMEM182*; Pearson’s r = 0.33, *p* = 1e-13, for *BARX2*; Pearson’s r = 0.5, *p* = 0, for *BCL9*; Pearson’s r = 0.49, *p* = 0, for *KCNK1*) and LINC00467 in LUAD are presented in **Figure 7C**. Ultimately, we constructed the LINC00467- mediated ceRNA network of LUAD, including two miRNAs (hsa-miR-1225-5p, hsa-miR-575) and five mRNAs (*BARX2*, *BCL9*, *KCNK1*, *KIAA1324*, *TMEM182*) (**Figure 7D**). Total 7 potential binding sites between LINC00467 and hsa-miR-575 (five sites) or hsa-miR-1225-5p (two sites) were identified (**Supplementary Table 2**). To determine which candidate genes had an impact on the overall survival of LUAD patients, Kaplan-Meier curves were plotted in both the TCGA and meta-Gene Expression Omnibus (GEO) LUAD cohorts. We found that higher expression of *BARX2* (HR=1.56, Logrank *p=*0.0034 for TCGA cohort; HR=1.56, Logrank *p=*0.00045 for GEO cohort) and *BCL9* (HR=1.35, Logrank *p=*0.045 for TCGA cohort; HR=1.46, Logrank *p=*0.0038 for GEO cohort) correlated with poorer overall survival (**Figures 7E, F** **)**. These results suggest that *BARX2* and *BCL9* are promising potential prognostic biomarkers for LUAD patients.

## Discussion

Despite its high incidence and mortality, the exact cause of the development of LUAD is not fully understood. Considering that only about 1.5% of the human genome encodes proteins, and most of the genome is transcribed, non-coding RNAs such as lncRNAs may play crucial roles in biological processes (7). Researchers have increasingly found that lncRNAs play an important role in tumor biology (5, 32). Herein, we investigated the role of LINC00467 in LUAD and demonstrated that DNA copy amplification and hypomethylation were two vital mechanisms of LINC00467 upregulation and were associated with poor prognosis. We also constructed a putative ceRNA network of LINC00467 in LUAD *via* sponging multiple miRNAs and mRNAs. Although some of these findings lack additional experimental verification, our data indicate that LINC00467 may be a promising target and diagnostic or therapeutic biomarker for LUAD.

Previously, several studies have reported that LINC00467 may serve as a promotor in LUAD (17–21). All these studies have suggested that LINC00467 expression is elevated in both patients and LUAD cell lines and that increased LINC00467 expression indicates a poor prognosis. Among these studies, only one has focused on the upstream molecular mechanism of LIN00467 upregulation in LUAD, by suggesting a crucial role of signal transducer and activator of transcription 1 (STAT1) on transcriptional activation (20). In this study, we focused on the genetic or epigenetic alterations regulating LIN00467 expression. By analyzing TCGA data, we found that the genomic copy number of LIN00467 was increased, which predicted poor prognosis along with increased immune cell infiltration in LUAD. Furthermore, hypomethylation of the LIN00467 promoter was associated with its elevated expression in tumor tissues. In an *in vitro* study, we demonstrated that LIN00467 upregulation was mediated by DNA demethylation that drives lung cancer malignancy (33). Taken together, our data suggest two possible mechanisms of LIN00467 upregulation, thereby enriching our understanding of the molecular mechanisms of LIN00467 involved in LUAD progression.

The down-stream mechanisms of lncRNAs have also been widely investigated in cancer (7). ceRNAs are a common molecular regulatory mechanism of lncRNAs, and they have been intensively reported in different cancer types (6, 34, 35). According to the ceRNA hypothesis, lncRNAs can form a sponge with miRNAs to regulate the expression of target genes at the mRNA level (6). Recent studies have confirmed that ceRNAs have significant roles in cancer pathogenesis by altering the expression of key tumorigenic or tumor suppressive genes (6, 36–38). Two prior studies have investigated the ceRNA function of LIN00467 in LUAD (17, 18). However, these studies only focused on the interaction of LIN00467 with two or three miRNAs (miR-20b-5p, miR‐4779, and miR‐7978). A comprehensive miRNA expression profile landscape is yet to be established. Here, we conducted a miRNA expression profile analysis in LINC00467-knocked-down A549 cells and control cells. Our data indicated that the miRNA profile of control cells was quite distinct from LINC00467-knocked-down cells, and the differentially expressed miRNAs mediated a comprehensive ceRNA network that was involved in multiple biological pathways related to carcinogenesis. In summary, our study provided novel insights for the underlying mechanism of LIN00467 in a ceRNA network.

Overall, our data provided possible mechanisms for the abnormal upregulation of LINC00467 and demonstrated that it may play a role in tumorigenesis in LUAD *via* a ceRNA regulatory network. These findings highlight the potential for LINC00467 in becoming a new diagnostic, prognostic, or therapeutic biomarker for LUAD patients. However, a limitation of this study is the restricted ability to experimentally validate our microarray data using alternative techniques. Therefore, future molecular biology experiments (e.g., luciferase reporter assays, Real-Time Quantitative PCR) are needed to validate our results. Anyway, our findings will serve as a useful reference resource for future studies.

## Data Availability Statement

The original contributions presented in the study are included in the article/**Supplementary Material**. Further inquiries can be directed to the corresponding author.

## Author Contributions

WW, HB, and GL conceived and planned the experiments. WW and HB carried out the experiments. HB and YL contributed to the interpretation of the results. GL took the lead in writing the manuscript. All authors provided critical feedback and helped shape the research, analysis and manuscript.

## Funding

This project was supported by the Scientific Research Project of Hunan Provincial Health Commission (Grant No. 20200548), Hunan Clinical Research Center for Chronic Kidney Disease (Grant No. 2019SK4009). Hunan Province Office of Education (Grant No. 21B0052), Scientific Research Projects of the Health Commission of Hunan Province (Grant No. 202203050006) and Young Doctor Fund of Hunan Provincial People’s Hospital (Grant No. BSJJ202104).

## Conflict of Interest

The authors declare that the research was conducted in the absence of any commercial or financial relationships that could be construed as a potential conflict of interest.

## Publisher’s Note

All claims expressed in this article are solely those of the authors and do not necessarily represent those of their affiliated organizations, or those of the publisher, the editors and the reviewers. Any product that may be evaluated in this article, or claim that may be made by its manufacturer, is not guaranteed or endorsed by the publisher.
