# Supplementary material for: LINC00467 Is Upregulated by DNA Copy Number Amplification and Hypomethylation and Shows ceRNA Potential in Lung Adenocarcinoma
Source: Front Endocrinol (Lausanne). 2022 Jan 13;12:802463. doi: 10.3389/fendo.2021.802463 (PMC8792904; doi:10.3389/fendo.2021.802463)
Supplement: Supplementary file 8 [file Table_8.docx]

**SUPPLEMENTAL MATERIALS AND METHODS**

**LINC00467 Expression is Upregulated by DNA Copy Number Amplification and Hypomethylation and Acts as a CeRNA in** **Lung Adenocarcinoma**

Wen Wang^1^, Hao Bo^2^, Yumei Liang^3, 4, 5^, Guoli Li^3, 4, 5, *^

^1^Department of Cardio-Thoracic Surgery, Hunan Provincial People’s Hospital (The first-affiliated hospital of Hunan normal university), Changsha, Hunan, P.R. China, China.

^2^Clinical Research Center for Reproduction and Genetics in Hunan Province, Reproductive and Genetic Hospital of CITIC-Xiangya, China.

^3^Department of Nephrology and Laboratory of Kidney Disease, Hunan Provincial People’s Hospital (The first-affiliated hospital of Hunan normal university), China.

^4^Changsha Clinical Research Center for Kidney Disease, China.

^5^Hunan Clinical Research Center for Chronic Kidney Disease, China.

*Corresponding author at: Department of Nephrology, Hunan Provincial People’s Hospital (The first-affiliated hospital of Hunan normal university), No. 61# Jiefang West Road, Changsha 410005, Hunan, P.R. China

E-mail address: ttly2013@hunnu.edu.cn.

**Working sheets of raw data obtained from publicly available datasets**

1. Date presented in **Figures 2A, B, C** were analyzed by TIMER2.0 (<http://timer.cistrome.org/>).

2. Date presented in **Figures 2D, E, F and Figure 7B, C** were analyzed by GEPIA2 (<http://gepia2.cancer-pku.cn/#index>).

3. Date presented in **Figures 7 E, F** were analyzed by Kaplan-Meier Plotter：(<http://kmplot.com/analysis/index.php?p=background>).

4. The TCGA data analyzed in this study were obtained from PanCancer Atlas-LUAD cohort and GEO data analyzed in this study were obtained from GSE14814; GSE31908; GSE29013; GSE30219; GSE19188; GSE3141; GSE31210; GSE50081; GSE37745.
